# Microglial Activation in the Pathogenesis of Huntington’s Disease

**DOI:** 10.3389/fnagi.2017.00193

**Published:** 2017-06-19

**Authors:** Hui-Ming Yang, Su Yang, Shan-Shan Huang, Bei-Sha Tang, Ji-Feng Guo

**Affiliations:** ^1^Department of Neurology, Xiangya Hospital, Central South UniversityChangsha, China; ^2^Department of Human Genetics, Emory University School of Medicine, AtlantaGA, United States; ^3^Department of Neurology, Tongji Hospital, Huazhong University of Science and TechnologyWuhan, China; ^4^Key Laboratory of Hunan Province in Neurodegenerative Disorders, Central South UniversityChangsha, China; ^5^State Key Laboratory of Medical GeneticsChangsha, China; ^6^National Clinical Research Center for Geriatric DiseasesChangsha, China

**Keywords:** Huntington’s disease, microglia, microglial activation, M1 phenotype, M2 phenotype, pathogenesis

## Abstract

Huntington’s disease (HD) is an autosomal dominantly inherited neurodegenerative disorder caused by expanded CAG trinucleotide repeats (>36) in exon 1 of *HTT* gene that encodes huntingtin protein. Although HD is characterized by a predominant loss of neurons in the striatum and cortex, previous studies point to a critical role of aberrant accumulation of mutant huntingtin in microglia that contributes to the progressive neurodegeneration in HD, through both cell-autonomous and non-cell-autonomous mechanisms. Microglia are resident immune cells in the central nervous system (CNS), which function to surveil the microenvironment at a quiescent state. In response to various pro-inflammatory stimuli, microglia become activated and undergo two separate phases (M1 and M2 phenotype), which release pro-inflammatory cytokines (IL-1β, IL-6, and TNF-α), anti-inflammatory cytokines, and growth factors (TGF-β, CD206, and Arg1), respectively. Immunoregulation by microglial activation could be either neurotoxic or neuroprotective. In this review, we summarized current understanding about microglial activation in the pathogenesis and progression of HD, with a primary focus of M1 and M2 phenotype of activated microglia and their corresponding signaling pathways.

## Introduction

Huntington’s disease (HD) is a devastating neurodegenerative disease characterized by symptoms of cognitive disorder, motor impairment and mental disturbance. The prevalence of HD is 4–10 per 100, 000 in the western population, and the mean age of onset is 40 years old (Ross and Tabrizi, 2011). It is an autosomal dominant monogenic disease caused by CAG trinucleotide repeats expansion in the *HTT* gene, which translates into huntingtin (HTT) protein with an expanded polyglutamine (polyQ) tract that is prone to misfolding. Although the pathogenic mutant huntingtin (mHTT) is ubiquitously expressed in different types of neural cells in the brain (Jansen et al., 2017), it causes a preferential loss of medium spiny neurons (MSNs) in the striatum (Graveland et al., 1985), and the atrophy of caudate and putamen could be clinically imaged by magnetic resonance imaging (MRI) prior to motor symptom onset (Bates et al., 2015).

Neuroinflammation, characterized by remarkable gliosis and inflammatory reactions in the central nervous system (CNS), has been described as a prominent sign in various neurodegenerative diseases such as Alzheimer’s disease (AD) (Villegas-Llerena et al., 2016), Parkinson’s disease (PD) (Le et al., 2016), and Amyotrophic Lateral Sclerosis (ALS) (Philips and Robberecht, 2011). HD is no exception, as mounting evidence indicates that microglia activation could be detected in the brains from pre-symptomatic HD carriers to post-mortem HD patients. Specifically, elevated inflammatory cytokines could be detected in both the CNS and plasma from HD patients (Tai et al., 2007a,b; Bjorkqvist et al., 2008; Silvestroni et al., 2009; Politis et al., 2015).

Microglia, the resident macrophage of the CNS, monitor the microenvironment in the CNS and respond to neural damage or degeneration by switching to different activation states (Hanisch and Kettenmann, 2007). Moreover, CX3CL1-CX3CR1 and CD200-CD200R are two major signaling pathways that function to mediate neuron-microglia interaction and facilitate immunomodulatory and phagocytic activities of microglia in response to degenerated neurons in the CNS (Gomez-Nicola and Perry, 2015). Under normal conditions, resting microglia typically display small cell bodies with fine processes and work as the first line of defense in the CNS (Hanisch and Kettenmann, 2007). In the presence of adverse stimuli, microglia retract the processes and exhibit swollen shapes (Moller, 2010). Microglia exhibiting large amoeboid-like cell bodies with no processes or with short stout extensions were seen in both HD patient brain (Sapp et al., 2001) and YAC128 HD mouse model (Franciosi et al., 2012). In addition, the abnormal morphology of microglia is exacerbated in an age-dependent manner, throughout the course of HD progression. Here, we review the immune-regulatory role of microglia in HD pathology, and discuss potential cellular mechanisms of microglia activation underlying HD pathogenesis.

## Pathogenic mHTT and Experimental Models for HD

Expanded polyQ tract at the N-terminus of HTT is responsible for protein misfolding and the subsequent aberrant accumulation and aggregates formation. Aggregated mHTT could be detected as inclusion bodies either in the cytoplasm or nuclei (DiFiglia et al., 1997; Gutekunst et al., 1999). The relative contribution of soluble and aggregated forms of mHTT to the pathogenesis in HD is still unclear. Nevertheless, it is commonly believed that the expression of N-terminal mHTT fragments could lead to HD pathogenesis, and the sizes of the fragments correlate inversely with neurotoxicity (Mangiarini et al., 1996; Chang et al., 2015).

To facilitate investigation of HD, a variety of HD experimental models with the expression of either full-length or N-terminal fragments of mHTT were generated and studied, such as transgenic animal models (Chang et al., 2015), human embryonic stem cell derived models (Lu and Palacino, 2013) and HD patients derived induced pluripotent stem cells (Xu et al., 2017). Among them, mouse model is one of the most commonly used, due to its relative ease of genetic manipulation and physiological resemblance to human. In **Table 1**, we provide a summary of most commonly studied HD mouse models, in terms of their genetic background and mHTT expression pattern and level.

**Table 1:** Mouse models of Huntington’s disease.

Mouse	Types		Poly-Q	Protein	Protein	Protein expression	
model	of model	Promoter	repeat	context	expression level	in types of neural cell	Reference
R6/2	Transgenic	Human HTT	150 CAG	Human N-terminal 1–82 a.a.	75%	All	Mangiarini et al., 1996
N171-82Q	Transgenic	Murine prion	82 CAG	Human N-terminal 1-171 a.a.	20%	Neurons	Schilling et al., 1999
Hdh^Q111^	Knock-in	Murine Hdh	111 CAG	Murine full-length Hdh	50% or 100%	All	Wheeler et al., 1999
Q140	Knock-in	Murine Hdh	140 CAG	Murine full-length Hdh	50% or 100%	All	Menalled et al., 2003
HdhQ150	Knock-in	Murine Hdh	150 CAG	Murine full-length Hdh	100%	All	Heng et al., 2007
zQ175	Knock-in	Murine Hdh	175 CAG	Murine full-length Hdh	100%	All	Menalled et al., 2012; Southwell et al., 2016
YAC128	Transgenic	Human *HTT*	128 CAG	Human full-length mHTT	75%	All	Hodgson et al., 1999
BACHD	Transgenic	Human *HTT*	97 CAA/CAG	Human full-length mHTT	150%	All	Gray et al., 2008

Although mHTT is ubiquitously expressed, HD elicits selective cortical and striatal neurodegeneration. To understand mechanisms underlying the preferential neuronal vulnerability, mouse models that express mHTT in selective cell populations were generated using the Cre-Loxp system. For instance, transgenic mice carrying Cre recombinase with different promoters, such as Emx1 or Rgs9, were crossed with BACHD mice, allowing restricted expression of mHTT in the cortex and striatum, respectively (Wang et al., 2014). Similarly, mice that express exon1 of mHTT in neurons or only in cortical pyramidal neurons were created by crossing RosaHD mice with Nestin-Cre mice or Emx1-Cre mice (Gu et al., 2005). In addition, there are transgenic mice that selectively express mHTT in astrocytes (Bradford et al., 2009) or oligodendrocytes (Huang et al., 2015), which demonstrate the mHTT expression in non-neuronal cells also contributes to the pathogenesis of HD. Mice that express mHTT specifically in microglia have been generated by crossing RosaHD with Cx3cr1-Cre miceD (Crotti et al., 2014).

## Microglial Activation in HD

Marked astrogliosis and microgliosis were observed in the post-mortem brains of HD patients but not in normal control brains (Singhrao et al., 1999). It was reported that microglia accumulated in all grades of HD patients’ brains, and the density correlated to the degree of neuronal loss (Sapp et al., 2001). Indeed, *in vivo* positron emission tomography (PET) studies revealed that microglial activation was significant in affected HD brain regions and this pathology was more pronounced in more severe cases of HD (Pavese et al., 2006; Politis et al., 2011). Furthermore, PET also detected microglial activation in HD patients prior to disease manifestations (Tai et al., 2007b). These studies provide strong evidence indicating that microglial activation is an integral part of HD pathogenesis. Nonetheless, whether the activated microglia plays a protective or detrimental role in HD pathogenesis remains to be illustrated.

### Reactive Microglial Activation

Although mHTT is widely expressed, MSNs in the striatum are the most susceptible cell type to mHTT-mediated neurotoxicity. Furthermore, mHTT is more abundantly accumulated in neuronal dendrites and nerve terminals than in soma and other cell types, and *in vitro* studies showed that mHTT preferentially formed aggregates along neuronal processes and axonal terminals (Li et al., 2000; Zhao et al., 2016). Microglia, as an active sensor in the CNS, transform to activated states in response to pathological changes in the CNS (Hanisch and Kettenmann, 2007). In cortico-striatal brain slice and primary neuronal culture models, neuronal expression of mHTT initiates a local response of microglia, resulting in elevated numbers and activated morphological phenotypes. In particular, these proliferative microglia were prone to position along irregular neurites, but did not directly contributed to neuronal degeneration (Kraft et al., 2012). Over-activated microglia were demonstrated to be neurotoxic, due to their capacity to release toxins. Therefore, decreased number of reactive microglia coupled with downregulation of inflammatory cytokines was viewed as an indicator of pathologic alleviation in HD (Wang et al., 2014; Ochaba et al., 2016), and therapeutic methods aimed at lowering neuronal mHTT expression led to drastic amelioration of concomitant microglia activation (Wang et al., 2014). On the other hand, adding exogenous primary microglia to mHTT-expressing neurons increased neuronal survival, and this effect was proportional to the amount of microglia added (Kraft et al., 2012). Moreover, a recent *in vivo* study showed supplementing normal human glia to transgenic R6/2 HD mice exhibited neuronal protection as well as phenotypic improvement (Benraiss et al., 2016). The above evidence indicates that normal microglia can rescue mHTT-expressing neurons. Nonetheless, whether mHTT expression in microglia results in its phagocytic or immunoregulatory dysfunction, and how this contributes to HD neuropathology remain to be investigated.

### Cell-Autonomous Microglial Activation

Although mHTT is ubiquitously expressed in all types of cells, the frequency of inclusion bodies was observed at a much lower rate in microglia compared to others (Jansen et al., 2017), probably owing to the increased immunoproteasome subunits (Orre et al., 2013) and autophagosomes (Su et al., 2016), which renders microglia a higher capacity to degrade mHTT. In spite of less mHTT aggregates formed in microglia, a cell autonomous effect induced by intrinsic mutant protein may be responsible for microglia activation and contribute to the pathology in HD as well. It was demonstrated that mHTT expression altered the function of immune cells both centrally and peripherally, and isolated microglia from R6/2 mice were described to be much more hyperactive than wild type microglia in response to stimulation (Bjorkqvist et al., 2008). Intriguingly, using genome-wide approaches, expression of mHTT in microglia was found to promote cell-autonomous pro-inflammatory gene expression in the absence of sterile inflammation and is dependent on the expression and transcriptional activities of the myeloid lineage-determining factors PU.1 and C/EBPs. In addition, mHTT-expressing microglia-mediated cell-autonomous activation exhibits enhanced toxic effects on wild type neurons in comparison to wild type microglia *ex vivo* and after sterile inflammation *in vivo* (Crotti et al., 2014). However, whether restricting the expression of mHTT in microglia is sufficient to exert neuropathological and behavioral deficits *in vivo* still needs to be investigated.

### Interplay between Microglial Activation and Astrocytic Activation

Previous work suggests over-activation of *N*-methyl-D-aspartate receptor (NMDAR) by extracellular glutamate is involved in the degeneration of medium-sized spiny neurons in HD (Zeron et al., 2002). Astrocytes, the major type of glia in brain, express glutamate transporters that uptake extracellular glutamate while mHTT expression in astrocytes diminished this protection against glutamate neurotoxicity (Shin et al., 2005). Moreover, selective mHTT expression in astrocytes caused age-dependent neurological symptoms and exacerbated neuronal loss *in vivo* (Bradford et al., 2009, 2010). Markedly, reactive astrocytes are accumulated in proximity to degenerated neurons in HD brain, characterized by increased proliferation, cell hypertrophy and the induction of astroglial markers [e.g., glial fibrillary acidic protein (GFAP)]. Furthermore, activated astrocytes instead of microglia were shown to upregulate the pro-inflammation in N171-82 HD mice that express mHTT only in neurons, but not in glial cells (Hsiao et al., 2013). However, a recent study revealed that neurotoxic reactive astrocytes could be induced by activated microglia and consequently resulted in a similar expression profile of pro-inflammatory cytokines, which led to a more rapid death of neurons and oligodendrocytes (Liddelow et al., 2017). Herein, the relative contribution by activated astrocytes and activated microglia to neuroinflammation and the eventual HD pathogenesis remains to be elucidated.

## Implication of Functional Phenotypes of Microglia in HD

In response to neurodegeneration and the accumulation of misfolded proteins, microglia multiply and adopt a process referred to as priming. Priming makes the microglia susceptible to a secondary inflammatory stimulus, which then triggers an exaggerated inflammatory response (Perry and Holmes, 2014). As the main dynamic component of neuroinflammation in the CNS, activated microglia exist along a continuum of two functional states of polarization in which they are able to either expand the damage to neighboring cells, or clear the cell debris followed by tissue repair (Jha et al., 2016). In reference to peripheral macrophage polarization, microglia share comparative properties in reaction to acute or prolonged stimuli, and are classified into two extreme phenotypes: the classically activated M1 phenotype and the alternatively activated M2 phenotype. Differences between these two phenotypes range from morphological changes to alteration of representative cytokines, determined by protein or gene expression profiling (Durafourt et al., 2012; Hu et al., 2015). To modify this simplified nomenclature, there are other proposals of classifying macrophages into more informative populations (Mosser and Edwards, 2008) or refining M2 categorization further into alternative activation (M2a), type II alternative activation (M2b) and acquired deactivation (M2c) (Walker and Lue, 2015). Yet the existence of these refined cell populations has not been fully examined in HD brain.

### M1 Phenotype of Microglia and Implicated Biomarkers in HD

When triggered by extracellular or intracellular stimuli, resident microglia conferred classically activated state named M1 phenotype to initiate and augment the innate immune function in the CNS. Classically activated M1 microglia release pro-inflammatory biomarkers such as redox molecules (NO, iNOS), cytokines (IL-1β, IL-6, IL-8, and TNF-α et al.), chemokines (CCL2, CCL20, and CXCL-1 et al.) and surface receptors (CD16, CD32, CD36, CD68, and CD86 et al.) which can be used to identify M1 phenotype of microglia (Franco and Fernandez-Suarez, 2015; Orihuela et al., 2016). *In vitro* and *in vivo* studies declared that M1 microglia could be induced by the treatment of LPS alone, or combining LPS/IFN-γ followed by increased levels of pro-inflammatory cytokines (Durafourt et al., 2012; Kroner et al., 2014; Cunha et al., 2016; Wang et al., 2016). M1 microglia relevant biomarkers were widely detected in HD brain, which indicates that M1 microglia may play a crucial role in the pathogenesis of HD. Primary glia cells including microglia, isolated from R6/2 transgenic mouse model, showed that iNOS, IL-1β, IL-6, and TNF-α were significantly elevated after LPS treatment (Hsiao et al., 2014), and IL-6 was increased in the microglia of R6/2 mouse brain (Bjorkqvist et al., 2008). In addition, higher levels of IL-1β and IL-8 were secreted by microglia in HD transgenic porcine model (Valekova et al., 2016). Furthermore, pro-inflammatory cytokines IL-1β, IL-6, IL-8, and TNF-α were elevated both centrally (in the striatum and cerebrospinal fluid) and peripherally (in the plasma) in HD patients (Bjorkqvist et al., 2008; Chang et al., 2015; Rodrigues et al., 2016). M1 related markers involved in HD were summarized as **Table 2**.

**Table 2:** Implications of M1 and M2 microglia relevant markers in HD.

M1 marker	Description	Reference
IL-1 β	Pro-inflammatory cytokine	Primary microglia isolated from transgenic HD porcine model (Valekova et al., 2016) Plasma of HD patient (Politis et al., 2015)
IL-6		Primary glial cells isolated from R6/2; Plasma and CSF from HD patient (Bjorkqvist et al., 2008) Plasma of HD patient (Dalrymple et al., 2007; Politis et al., 2015) Post-mortem brain of HD patient (Silvestroni et al., 2009) Plasma of HD patient and mouse model (Chang et al., 2015)
IL-8		Primary microglia isolated from transgenic HD porcine model (Valekova et al., 2016) Primary glial cells isolated from R6/2; Plasma and CSF from HD patient (Bjorkqvist et al., 2008) Plasma of HD patient (Politis et al., 2015) Post-mortem brain of HD patient (Silvestroni et al., 2009)
TNF-α		Primary glial cells isolated from R6/2; Plasma and CSF from HD patient (Bjorkqvist et al., 2008) Plasma of HD patient (Politis et al., 2015)
CCL2	Chemokine	Post-mortem brain of HD patient (Silvestroni et al., 2009)
MMP-9	Extracellular proteins	Post-mortem brain of HD patient (Silvestroni et al., 2009) Plasma of HD patient and mouse model (Chang et al., 2015)
M2 Marker	Description	Reference
IL-10	Cytokine	Post-mortem brain of HD patient (Silvestroni et al., 2009)
VEGF	Growth factor	Plasma of HD patient and mouse model (Chang et al., 2015)
TGF-β		(Di Pardo et al., 2013) Plasma of HD patient and mouse model (Chang et al., 2015)
IGF-1		Primary microglia isolated from transgenic HD porcine model (Valdeolivas et al., 2015)

*IL, interleukin; TNF-α, tumor necrosis factor alpha; chemokine CCL2, C-C motif ligand 2; MMP-9, matrix metallopeptidase 9; VEGF, vascular endothelial growth factor; TGF-β, transforming growth factor beta; IGF-1, insulin-like growth factor 1.*

### M2 Phenotype of Microglia and Implicated Markers in HD

In contrast to M1 microglia, alternative activated M2 microglia enable phagocytosis and initiate tissue repair and neural regeneration in the CNS (Miron et al., 2013). M2 microglia are characterized by producing anti-inflammatory cytokines and growth factors such as IL-10, TGF-β, CD206, Arg1, Ym1 (Chi3l3 in human) and Fizz1 (Franco and Fernandez-Suarez, 2015; Orihuela et al., 2016). Of these molecules, IL-10 played a key role in phagocytic microglia to engulf apoptotic cells (Chhor et al., 2013; Cianciulli et al., 2015). CD206, Arg1, and Ym1 were considered as typical markers for the identification of M2 microglia (Chhor et al., 2013; Miron et al., 2013; Zanier et al., 2014). To recapitulate the phenotype of M2 microglia, manipulation of IL-10 alone or combining IL-4/IL-13 was performed to establish the experimental models (Ponomarev et al., 2007; Chhor et al., 2013; Miron et al., 2013). Much less M2 markers were found in HD, as only VEGF and TGF-β were reported to coexist with M1 markers in the plasma and post-mortem brain tissues of HD patients (Di Pardo et al., 2013; Chang et al., 2015). M2 related markers in HD were summarized as **Table 2**.

### Concurrence and Transformation of M1 and M2 Microglia

As illustrated above, M1 and M2 microglia are defined by their contradictory functions and distinctive biomarkers. However, in traumatic brain injury (TBI), up-regulated expression of both M1 and M2 microglia associated genes can be found simultaneously. And co-localization of representative biomarkers of both activation states in the same microglia indicated concurrence of M1 and M2 phenotype following TBI (Morganti et al., 2016). Consistent phenomenon of concurrent M1/M2 response was found in ALS (Chiu et al., 2013) and spinal cord injury (SCI) (Shechter et al., 2013) as well. In addition, M1 microglia are able to transform to M2 microglia, as cyclic adenosine monophosphate (cAMP) has been reported to play a key role in converting LPS/IFN-γ induced M1 microglia to M2 microglia, which augments the anti-inflammatory effects in the presence of IL-4 with increased levels of Arg1 (Ghosh et al., 2016). In addition, switching M1 microglia to M2 microglia was demonstrated to be protective by initiating remyelination in multiple sclerosis (MS) (Miron et al., 2013). Concurrent inflammatory profiles of M1 and M2 microglia related biomarkers were also indicated in HD as shown as **Table 2**.

## Signaling Pathways Underlying the Microglial Activation in HD

### Transcriptional Factor Nuclear Factor Kappa B (NF-KB) Pathway

Microglia express toll like receptors (TLRs) to recognize extracellular stimuli. TLR-2 is the most expressed TLR in microglia, which is responsible for IL-6 and IL-10 secretion. TLR-3 and TLR-4 are two receptors, which most potently stimulate proinflammatory cytokines secretion, such as IL-12, TNF-α, IL-6, CXCL-10, and IL-10 (Jack et al., 2005). Studies showed that TLR-2, TLR-3, and TLR-4 activate microglia and astrocytes in a sequential manner, coupled with elevated IL-1β level (Facci et al., 2014). Experimental M1 microglia, obtained by the treatment of LPS or LPS/IFN-γ, were capable to increase TLR-2 and TLR-4 gene expression (Marinelli et al., 2015). Classical TLR signaling involves the intracellular adaptor protein myeloid differentiated 88 (MyD88) to trigger downstream NF-κB signaling cascade and thus upregulates the production of proinflammatory cytokines (Takeda and Akira, 2004). NF-κB is sequestered in the cytoplasm by I-κBs, a group of inhibitory proteins (Ghosh et al., 1998). When I-κBs are phosphorylated by I-κB kinase, NF-κB is dissociated from I-κBs and translocated to the nucleus (IKK including subunits of IKKα, IKKβ, and IKKγ) (Ghosh and Karin, 2002). Consequently, NF-κB regulates downstream inflammatory cytokines gene expression. Soluble mHTT was shown to activate IKK, which in turn upregulates the NF-κB signaling pathway (Khoshnan et al., 2004). Trager et al. used proximity ligation assays and offered evidence that mHTT interacted with IKK, which also mediated transcriptional changes of NF-κB signaling with increased levels of IL-1β, IL-6, IL-8, and TNFα. Furthermore, lowering mHTT level by siRNA was shown to ameliorate NF-κB transcriptional dysregulation, and downregulate the expression of pro-inflammatory cytokines in HD (Trager et al., 2014).

### Kynurenine Pathway (KP)

In mammalian cells, Kynurenine pathway (KP) mediates degradation of the majority of cellular tryptophan through different enzyme branches (Fujigaki et al., 2017). The 3-hydroxyanthranilate 3, 4-dioxygenase (KMO) enzyme, which is predominantly expressed in microglia, metabolizes tryptophan to neuroactive 3-hydroxykynurenine (3HK) and quinolinic acid (QUIN) (Heyes et al., 1992). QUIN could lead to neurotoxicity by acting as a selective agonist on NMDA receptors (Szalardy et al., 2012), and it has been associated with neuroinflammation in various neurological diseases (Maddison and Giorgini, 2015). Through a genome wide screening in yeast, *BNA1*, which encodes the enzyme Bna1 [3, 4-dioxygenase (KMO)] involved in the kynurenine pathway, was found to mediate mHTT toxicity by producing high levels of QUIN and 3HK (Giorgini et al., 2005), which activate NMDA receptors and generate toxic free radical, respectively (Schwarcz and Pellicciari, 2002). However, the first and rate-limiting enzyme of the kynurenine pathway, indoleamine 2,3-dioxygenase (IDO), preferentially localizes in microglia (Heyes et al., 1996), but not in astrocytes or neurons of human brain (Guillemin et al., 2005). A recent study using transgenic N171-82Q HD mouse model revealed that IDO activity was significantly elevated in HD mice compared to wild type control mice (Donley et al., 2016). Moreover, previous studies (Guidetti et al., 2004, 2006) revealed that IDO activity was elevated in the early stage of HD, which contributed to increased levels of HK and QUIN- and NMD-mediated neurotoxicity. Taking into account of the studies above, drugs that can inhibit KP enzymes may offer potential therapeutic approaches by preventing neurotoxicity caused by downstream toxic products (Schwarcz and Pellicciari, 2002). As KMO and QUIN production showed cell type specific expression in microglia, chronic administration of KMO inhibitor JM6 was shown to prevent synaptic degeneration and increase the survival of R6/2 HD mouse model, which is associated with amelioration of microglial activation (Zwilling et al., 2011). In addition, suppressing the expression of tryptophan-2, 3-dioxygenase (TDO), another rate limiting enzyme in KP, was demonstrated to be neuroprotective in MS (Lanz et al., 2017). Additionally, inhibiting KP is likely to be protective in several neurologic diseases (Fujigaki et al., 2017), probably by modulating microglial activation mediated neurotoxicity.

### Cannabinoid (CB) Receptors Signaling

Cannabinoid receptors are a group of G-protein coupled receptors including cannabinoid type 1 (CB_1_) receptors and cannabinoid type 2 (CB_2_) receptors (Glass and Northup, 1999). In particular, CB_2_ receptors were involved in regulating cell proliferation, differentiation and survival through several signaling pathways, which include adenylyl cyclase and cyclic AMP-protein kinase A (PKA), extracellular signal-regulated kinase 1 (ERK1) and ERK2, p38 mitogen-activated protein kinase and JUN N-terminal kinases (JNKs) (Bisogno et al., 2016). In R6/2 HD mouse model, CB_1_ receptors were abundantly expressed in striatal MSNs (Chiarlone et al., 2014) and exerted a neuroprotective role through induction of brain-derived neurotrophic factor (BDNF) expression (Blazquez et al., 2015). CB_2_ receptors were reported to be mainly expressed in periphery immune cells, and were barely detected in brain tissue (Schatz et al., 1997). However, a significant increase of CB_2_ receptors expression was seen in traumatic mouse brain (Donat et al., 2014) and in activated microglia in AD mouse model (Benito et al., 2003). In addition, activation of CB_2_ receptors in microglia was shown to influence the acquisition of M2 polarization, and this effect was dampened by knocking out CB_2_ receptors (Mecha et al., 2015). As for HD, a study showed genetic ablation of CB_2_ receptors in R6/2 mouse model exacerbated the behavioral abnormalities and reduced life span, along with accelerated microglial activation (Palazuelos et al., 2009). The further impairment in motor activities caused by loss of CB_2_ receptors was recapitulated in BACHD mouse model as well (Bouchard et al., 2012). Therefore, therapeutic approaches targeting CB_2_ receptors in microglia might be promising in treating HD. Indeed, R6/2 mice have been treated with cannabigerol (CBG), a non-psychotropic phytocannabinoid that exerted neuroprotective effects through both CB_1_/CB_2_ receptors-dependent and -independent mechanisms. Administration of CBG significantly improved motor impairment and increased the expression of BDNF and insulin-like growth factor-1 (IGF-1) in treated R6/2 mouse model (Valdeolivas et al., 2015). In addition, VCE-003, a CBG quinone derivative, also attenuated striatal neuron loss, motor impairment and microglial activation in quinolinic acid (QA) and 3-NP induced HD mouse models (Diaz-Alonso et al., 2016). A CB_2_ receptor specific agonist, SR144528, has also been used for treating mice, which reduced the generation of proinflammatory molecules such as TNF-α (Sagredo et al., 2009).

## Conclusion and Future Directions

Microglial activation has been found for over a decade in HD brain and indicated as a strong component of neuroinflammation in HD pathogenesis (Crotti and Glass, 2015). Microglial activation in HD was reported to be triggered by neuronal mHTT-mediated excitotoxicity or mHTT expression in microglia, however, whether microglial activation makes a significant contribution to HD pathology has not been fully investigated. Polarized M1 phenotype and M2 phenotype of microglia, which have been illustrated in neurological diseases, especially traumatic brain injury (Morganti et al., 2016) or SCI (Kroner et al., 2014), exert coherent morphologic and functional features with alteration of central or peripheral inflammatory profiles. Although M1 and M2 microglia relevant biomarkers are found in HD, the definition and identification of these polarized phenotypes of microglia in HD remain to be elucidated. It is possible that microglial activation play dual roles in HD, either detrimental or beneficial, and thus targeting signaling pathways specific in protective properties of microglia may offer potential therapeutics.

## Author Contributions

H-MY conceived the study and wrote the manuscript. H-MY, SY, S-SH, B-ST, and J-FG discussed and revised the manuscript. H-MY prepared the tables. All authors read and approved the final version of the manuscript.

## Conflict of Interest Statement

The authors declare that the research was conducted in the absence of any commercial or financial relationships that could be construed as a potential conflict of interest. The reviewer EP-N and handling Editor declared their shared affiliation, and the handling Editor states that the process nevertheless met the standards of a fair and objective review.
